# A SIRT1-stabilizing nanofibrous platform via suppression of ubiquitination for enhanced diabetic wound healing: synergistic therapy with saracatinib and MOF-818

**DOI:** 10.1186/s12951-026-04663-0

**Published:** 2026-06-16

**Authors:** Pingping Xue, Zhi Yin, Yuhang Chen, Anran Zhang, Chenyu Zhuo, Hai Du, Nuo Xu, Renqun Mao, Xiaofan Yang

**Affiliations:** 1 Department of Pharmacy, Tongji Hospital, Tongji Medical College, Huazhong University of Science and Technology, Wuhan, 430030 China; 2 Department of Dermatology, The first Affiliated Hospital with Nanjing Medical University, Nanjing, 210029 China; 3 Chongqing Hospital of Jiangsu Province Hospital, Chongqing, 401420 China; 4 Chongqing Hospital of The first Affiliated Hospital with Nanjing Medical University, Chongqing, 401420 China; 5 Department of Hand Surgery, Union Hospital, Tongji Medical College, Huazhong University of Science and Technology, Wuhan, 430022 China; 6 Department of Hand and Foot Surgery, Shenzhen Nanshan People’s Hospital, Shenzhen, 518052 China; 7 Hubei Key Laboratory of Regenerative Medicine and Multi-disciplinary Translational Research, Huazhong University of Science and Technology, Wuhan, 430022 China

**Keywords:** Chronic diabetic wounds, Nanofiber, Oxidative stress, MOF, SIRT1

## Abstract

Chronic diabetic wounds, characterized by persistent oxidative stress, dysregulated inflammation, impaired angiogenesis, and susceptibility to bacterial infection, present a significant clinical challenge due to delayed tissue repair. This study reports the development of a multifunctional nanofibrous dressing, termed P-G^MOF−818@Sec^, engineered by incorporating saracatinib (Sec)-loaded MOF-818 into an electrospun gelatin/polycaprolactone (P-G) membrane. The P-G nanofibrous matrix provides essential wound adhesion, local retention, and mechanical support. MOF-818 confers reactive oxygen species (ROS)-responsive degradation, nanozyme-mimetic ROS scavenging capabilities, and intrinsic antibacterial activity. The platform exhibited H_2_O_2_ concentration-dependent release of Sec, confirming its pathophysiology-guided responsiveness to the diabetic wound microenvironment. The released Sec inhibited Fyn kinase-mediated ubiquitination of SIRT1, thereby stabilizing SIRT1 protein levels and activating the downstream SIRT1/FOXO3a/SOD2 antioxidant pathway. In vitro, P-G^MOF−818@Sec^ effectively reduced intracellular ROS, protected endothelial cells, fibroblasts, and keratinocytes from oxidative injury, and promoted cell migration, proliferation, and VEGF secretion. The dressing demonstrated broad-spectrum antibacterial activity against both antibiotic-sensitive and drug-resistant strains, including methicillin-resistant *Staphylococcus aureus* (MRSA) and multidrug-resistant *Escherichia coli* (MDR *E. coli*). In a diabetic rat wound model, P-G^MOF−818@Sec^ significantly accelerated wound closure, enhancing angiogenesis, collagen deposition, re-epithelialization, and facilitating a shift from M1 to M2 macrophage polarization. Comprehensive evaluations of mechanical properties, Cu/Zr biodistribution, and serum biochemistry supported the structural applicability and favorable preliminary biosafety of the platform. Collectively, P-G^MOF−818@Sec^ represents a pathophysiology-guided therapeutic strategy that uniquely integrates extracellular microenvironment regulation with intracellular stabilization of a key cytoprotective protein, SIRT1, for enhanced diabetic wound healing.

**Graphical abstract:**

**Figure:**
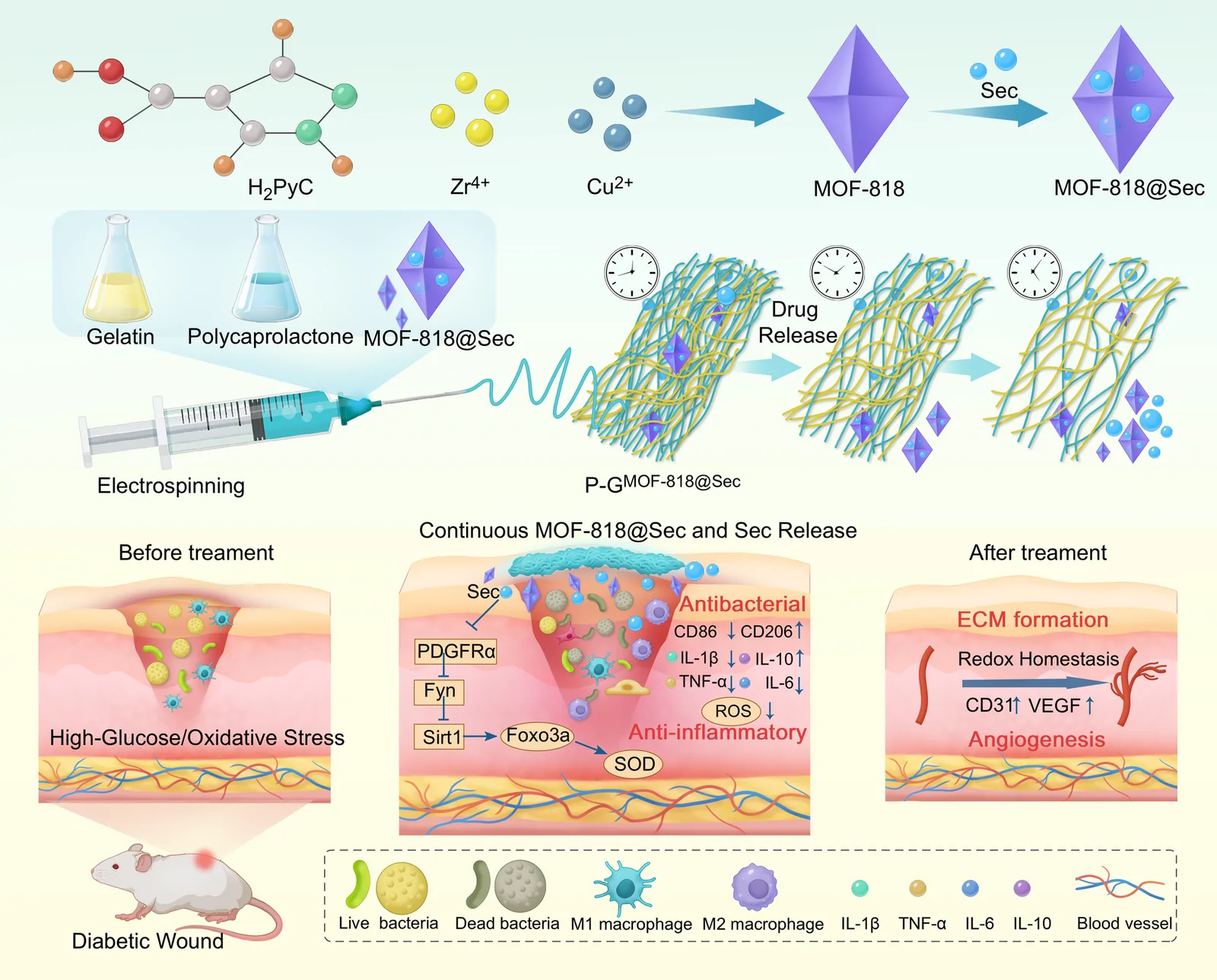

**Supplementary Information:**

The online version contains supplementary material available at https://doi.org/10.1186/s12951-026-04663-0.

## Introduction

Diabetes mellitus affects over 537 million adults globally, with its prevalence projected to rise continually by 2045 [1]. Diabetic foot ulcers (DFUs) are among the most severe chronic complications of diabetes, associated with persistent infection, delayed healing, high recurrence rates, amputation, and increased mortality [2–4]. The pathogenesis of DFUs is multifactorial, involving vascular dysfunction, immune dysregulation, and impaired functionality of keratinocytes, fibroblasts, and endothelial cells [2, 3]. Consequently, DFUs pose a growing public health challenge, necessitating advanced therapeutic strategies capable of simultaneously addressing the complex pathological wound microenvironment [4].

Hyperglycemia drives excessive accumulation of reactive oxygen species (ROS), overwhelming endogenous antioxidant defenses and leading to oxidative cellular damage that impedes tissue repair [5, 6]. This has spurred the development of ROS-regulating biomaterials and nanozyme-based platforms aimed at remodeling the oxidative diabetic wound microenvironment [7–10]. The SIRT1/FOXO3a signaling axis plays a pivotal role in cellular redox homeostasis. SIRT1 deacetylates FOXO3a, promoting the expression of antioxidant enzymes such as superoxide dismutase 2 (SOD2) and catalase (CAT) [11]. In diabetic models, modulation of SIRT1-related signaling has been shown to restore endothelial angiogenic function and accelerate wound closure, highlighting SIRT1 as a promising therapeutic target [12]. However, under diabetic oxidative stress, SIRT1 expression and activity are suppressed, and its degradation via the ubiquitin-proteasome pathway is enhanced, further weakening this critical cytoprotective signaling [13–15]. Concurrently, hyperglycemia impairs VEGF-mediated angiogenesis, while immune dysfunction and bacterial colonization exacerbate inflammation and hypoxia within the chronic wound [2, 16]. Conventional passive dressings are inadequate to counter these interconnected barriers, underscoring the urgent need for multifunctional systems capable of actively modulating the wound milieu [17].

Recent advances in biomaterial-based wound dressings, including bioactive hydrogels, microneedles, nanozyme systems, and responsive nanocomposites, have demonstrated potential in improving diabetic wound repair by targeting oxidative stress, infection, inflammation, angiogenesis, and tissue regeneration [7–10, 18–21]. For instance, immunomodulatory hydrogel systems that neutralize ROS and improve oxygenation can remodel the diabetic wound microenvironment and promote macrophage polarization toward a reparative phenotype [22]. Similarly, inherently antibacterial hydrogel dressings have been developed to suppress bacterial colonization and facilitate the healing of infected wounds [23]. These studies collectively emphasize that effective diabetic wound therapy requires coordinated intervention against multiple pathological factors rather than targeting a single element.

Electrospun nanofibers are promising candidates for advanced wound dressings due to their structural similarity to the native extracellular matrix (ECM) and tunable drug delivery capabilities [17, 24–27]. Blends of polycaprolactone and gelatin (P-G) offer favorable spinnability, biocompatibility, hydrophilicity, and mechanical integrity, forming fibrous scaffolds that support cell adhesion and tissue ingrowth [28–32]. Incorporating functional nanomaterials into such electrospun matrices can further confer targeted biological activities, addressing the multifactorial pathology of chronic wounds [26, 27, 30, 31]. Metal-organic frameworks (MOFs) have emerged as attractive nanoplatforms owing to their high surface area, porous architecture, stimuli-responsive degradation, intrinsic bioactivity, and nanozyme-mimetic catalytic potential [33–37]. Notably, MOF-818, a copper-based framework featuring trinuclear Cu clusters and a well-defined octahedral morphology, exhibits intrinsic superoxide dismutase (SOD)- and catalase (CAT)-like activities, enabling efficient ROS scavenging [38, 39]. Its responsiveness to oxidative environments allows for on-demand structural degradation and payload release, while the released Cu^2+^ ions may contribute to antibacterial activity under pathological wound conditions [40].

**Fig. 1:**
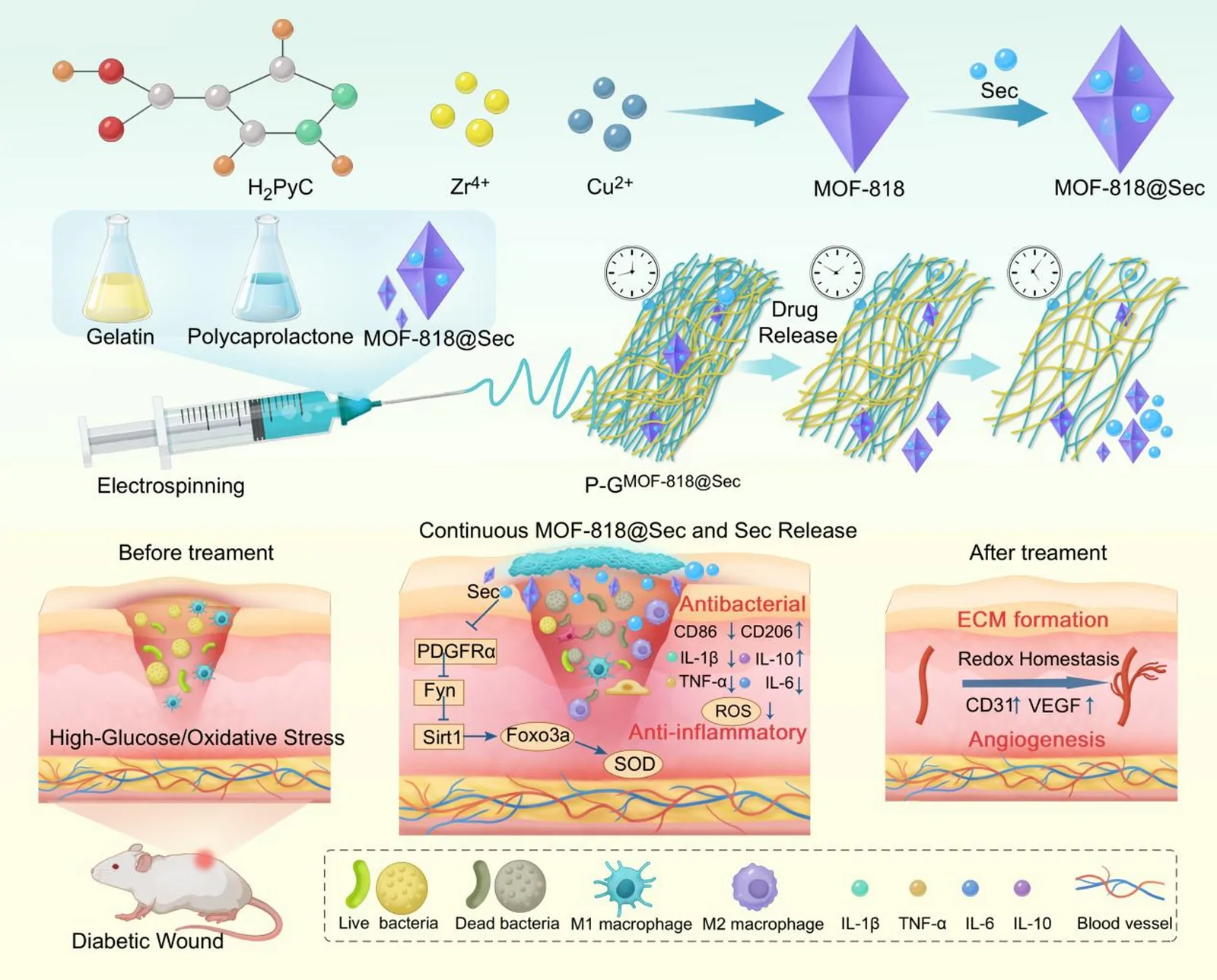
Illustration of the preparation of MOF-818 and P-G^MOF-818@Sec^ and their application in diabetic wound therapy

In this study, we introduce saracatinib (Sec), a clinical-stage Src/Abl kinase inhibitor with high potency against Fyn kinase [41]. Inhibition of Fyn is anticipated to suppress Fyn-associated ubiquitination and destabilization of SIRT1, thereby restoring the SIRT1/FOXO3a/SOD2 antioxidant signaling axis and strengthening endogenous cytoprotective responses [11–14]. By integrating Sec-loaded MOF-818 into a P-G nanofibrous membrane, we designed P-G^MOF-818@Sec^ as a pathophysiology-guided dressing for diabetic wound repair. In this integrated system, the P-G membrane provides a wound-conformal fibrous scaffold for local retention and sustained delivery; MOF-818 contributes ROS-responsive degradation, nanozyme-like ROS scavenging, and antibacterial activity; and the released Sec acts to stabilize SIRT1 and activate intracellular antioxidant signaling. Distinct from previous diabetic wound dressings that primarily rely on extracellular ROS scavenging, oxygen supplementation, antibacterial agents, or passive drug delivery [7–10, 18–23], P-G^MOF-818@Sec^ is designed to synergistically combine MOF-818-mediated extracellular microenvironment regulation with Sec-mediated intracellular SIRT1 stabilization. We hypothesized that this coordinated extracellular-intracellular strategy would simultaneously attenuate key pathological barriers in diabetic wound healing, including ROS accumulation, bacterial burden, inflammatory imbalance, impaired angiogenesis, and delayed tissue remodeling (Fig. 1).

## Materials and methods

### Materials

Zirconyl chloride octahydrate (ZrOCl_2_·8H_2_O), copper(II) nitrate trihydrate (Cu(NO_3_)_2_·3H_2_O), and pyridine-2,5-dicarboxylic acid (H_2_PyC) were purchased from Beijing Innochem Science & Technology Co., Ltd. Gelatin, trifluoroacetic acid (TFA), N,N-dimethylformamide (DMF), and poly(ε-caprolactone) (PCL, M_n_ = 80,000) were obtained from Shanghai Macklin Biochemical Technology Co., Ltd. Saracatinib was procured from TargetMol Chemicals Inc. Human umbilical vein endothelial cells (HUVECs) were acquired from Zhong Qiao Xin Zhou Biotechnology Co., Ltd. (China). The human keratinocyte cell line (HaCaT) and mouse fibroblast cell line (L929) were sourced from Shanghai Celliver Biotechnology Co., Ltd. Cell Counting Kit-8 (CCK-8) was from Abbkine Scientific Co., Ltd. Actin-Tracker Green-488 was from Beyotime Biotechnology (China). Primary antibodies against SOD2, SIRT1, and FOXO3a were purchased from Beijing Qinke Biotechnology Co., Ltd. and Beijing San Ying Biotechnology Co., Ltd.

### Preparation and characterization of MOF-818

MOF-818 was synthesized via a modified solvothermal method [39, 40]. Briefly, ZrOCl_2_·8H_2_O (42.5 mg), Cu(NO_3_)_2_·3H_2_O (124.0 mg), and H_2_PyC (2,5-pyridinedicarboxylic acid, 32.5 mg) were dissolved in 10 mL of N, N-dimethylformamide (DMF). The mixture was sonicated for 5 min, acidified with trifluoroacetic acid (TFA, 120 µL), and heated at 100 °C for 11 h in a Teflon-lined autoclave. After cooling, the resulting blue crystals were washed sequentially with DMF and acetone, with solvent changes three times daily for two days each. The product was then dried under vacuum at 60 °C for 5 h. Hydrodynamic size was measured by dynamic light scattering (DLS). Chemical structure was confirmed by Fourier-transform infrared spectroscopy (FT-IR), and crystallinity was analyzed by X-ray diffraction (XRD). Morphology and elemental composition were examined by transmission electron microscopy (TEM), scanning electron microscopy (SEM), and energy-dispersive X-ray spectroscopy (EDS). Porosity was evaluated by N_2_ adsorption-desorption analysis, and thermal stability was assessed by thermogravimetric analysis (TGA).

### Evaluation of the nanozyme activities of MOF-818

The superoxide anion (·O_2_^–^) scavenging activity of MOF-818 (10 mg/mL) was evaluated using electron paramagnetic resonance (EPR, Bruker EMXnano). The SOD- and CAT-like activities of MOF-818, MnO_2_, and Co_3_O_4_ (each at 1 mg/mL) were assessed following commercial kit protocols (Nanjing Jiancheng A007-1-1; Baisha BL903A), with catalase (20 U/mL) and SOD (224 U/mL) as positive controls. Oxygen generation kinetics were studied by adding MOF-818 (0–200 µg/mL) to 20 mM H_2_O_2_, with real-time dissolved oxygen monitored via a probe.

### Preparation and characterization of P-G^MOF−818@Sec^

PCL (1 g) and gelatin (200 mg) were dissolved in 10 mL of 1,1,1,3,3,3-hexafluoro-2-propanol (HFP) under stirring for 12 h to form a homogeneous P-G electrospinning solution. For P-G^MOF−818@Sec^, pre-formed MOF-818@Sec was uniformly dispersed in 2 mL of the P-G solution (75 mg/mL) under 30 min of stirring prior to electrospinning. The solutions were electrospun at an applied voltage of 14 kV and a flow rate of 2 mL/h to fabricate nanofibrous membranes. Membrane hydrophilicity was assessed by water contact angle measurement (Dataphysics OCA20). Fiber morphology and diameter distribution were analyzed by SEM (Hitachi SU8020) after gold sputter-coating.

Mechanical properties of P-G and P-G^MOF−818@Sec^ membranes were evaluated at room temperature using a universal testing machine (Instron 5965). Membrane strips (30 mm × 5 mm) were stretched at a constant crosshead speed of 5 mm/min until failure. Tensile strength, elongation at break, and Young’s modulus were derived from the stress–strain curves. For cyclic tensile testing, P-G and P-G^MOF−818@Sec^ membranes were subjected to three consecutive loading-unloading cycles at maximum strains of 10% and 35%, respectively, at the same crosshead speed, and structural integrity was recorded.

### The water vapor transmission rate (WVTR)

Circular P-G and P-G^MOF−818@Sec^ membranes (3 cm^2^) were mounted over Eppendorf tubes filled with deionized water. The assemblies were placed in a controlled environment (37 °C, 90% relative humidity) with pre-weighed CaCl_2_ (M_0_). After 3 h, the CaCl_2_ was re-weighed (M_1_), and the WVTR was calculated as: $\rm WVTR=(M1 - M0)/(Effective\,Film\,Area\times\,Time)$

### Drug loading capacity

The loading of Sec onto MOF-818 was quantified by UV-Vis spectroscopy (Shimadzu UV-2600) at 265 nm using a pre-established standard curve. MOF-818@Sect.  (1 mg/mL) was dispersed and measured under identical conditions. Encapsulation efficiency (EE) and drug loading capacity (DLC) were calculated using the equations: $$\begin{aligned}&\rm EE=(W_{loaded} / W_{initial}) \times 100\% ;\\& \,DLC =\,(W_{loaded}/W_{composite}) \times\,100\%\end{aligned}$$  

### In vitro drug release study

To evaluate ROS-concentration-dependent release, accurately weighed P-G^MOF−818@Sec^ membranes were immersed in phosphate-buffered saline (PBS) containing different concentrations of H_2_O_2_ (0, 10, 50, 100, and 200 µM) and incubated at 37 °C with gentle shaking. At predetermined time points, release media were collected and analyzed for Sec concentration by UV-Vis spectrophotometry at 265 nm. Cumulative release profiles were plotted. The release data were fitted to a zero-order kinetic model (C_t_ = C_0_ + kt), where C_t_ is the released Sec concentration at time t, C_0_ is the initial concentration, and k is the apparent zero-order release rate constant.

### Cytotoxicity assay

HUVECs, L929, and HaCaT cells were seeded in 96-well plates at 5 × 10^3^ cells/well. After overnight culture, cells were treated with Sec, MOF-818, or MOF-818@Sects.  (0–50 µg/mL) in medium containing 2% serum. After 48 h, cell viability was assessed using the CCK-8 kit according to the manufacturer’s instructions. Absorbance was measured at 450 nm using a microplate reader. Cell viability was calculated as follows:

$$\:\mathrm{C}\mathrm{e}\mathrm{l}\mathrm{l}\:\mathrm{V}\mathrm{i}\mathrm{a}\mathrm{b}\mathrm{i}\mathrm{l}\mathrm{i}\mathrm{t}\mathrm{y}\:\left({\%}\right)\:=\:\mathrm{(}{\mathrm{A}}_{\mathrm{t}}/{\mathrm{A}}_{\mathrm{c}})\times{100\%}$$

where Aₜ and A_c_ represent the absorbance of the test group and the control group (0 µg/mL), respectively.

### Antibacterial performance

Antibacterial activity was evaluated against methicillin-sensitive *S. aureus* (MSSA), methicillin-resistant *S. aureus* (MRSA), *E. coli*, and multidrug-resistant *E. coli* (MDR *E. coli*). Tested groups included Sec, MOF-818, MOF-818@Sec, and P-G^MOF−818@Sec^.

The minimum inhibitory concentration (MIC) was determined using a broth microdilution method in 96-well plates with two-fold serial dilutions (1–512 µg/mL) of the samples. Bacterial suspensions (~ 1.0 × 10^6^ CFU/mL) were added to each well. After incubation at 37 °C for 16–20 h, the MIC was defined as the lowest concentration that completely inhibited visible bacterial growth.

For colony counting, bacterial suspensions (~ 10^5^–10^7^ CFU/mL) were incubated with samples at 37 °C for 4–6 h, serially diluted, plated on LB agar, and incubated. Colony-forming units (CFUs) were counted. For Live/Dead staining, bacteria were treated, stained with DMAO and propidium iodide (PI), and imaged by fluorescence microscopy. The relative ratio of live bacteria was quantified based on green fluorescence intensity.

### Evaluation of antioxidant capacity in vitro

An oxidative stress cell model was established by co-treatment with high glucose and H_2_O_2_ to mimic the diabetic wound microenvironment. Cells were seeded and treated with glucose (10–140 mM) for 24 h to optimize the high-glucose concentration. Cells were then pre-incubated with the selected high-glucose medium for 24 h, followed by exposure to H_2_O_2_ (100–500 µM) for another 24 h to determine the appropriate oxidative dose. For protection assays, modeled cells were treated with PBS, Sec, MOF-818, MOF-818@Sec, or P-G^MOF−818@Sec^ for 6 h, then incubated with 2’,7’-dichlorodihydrofluorescein diacetate (DCFH-DA, 10 µM) for 30 min. Intracellular ROS levels were analyzed by fluorescence microscopy and flow cytometry.

### Cell viability staining

HUVECs, L929, and HaCaT cells were seeded in 24-well plates (4 × 10^4^ cells/well). After overnight culture, cells were treated with 80 mM glucose for 24 h, followed by 300 µM H_2_O_2_ for 6 h to induce oxidative injury. Subsequently, cells were incubated with the test samples for 48 h. For live/dead staining, cells were stained with Calcein-AM (2 µM) and propidium iodide (PI, 4 µM) for 20 min in the dark and imaged by fluorescence microscopy.

### Cell migration and proliferation assay

Cells were seeded in 24-well plates (1 × 10^5^ cells/well). After overnight culture, a high-glucose/oxidative stress (HG/OS) model was established as described (80 mM glucose for 24 h, then 300 µM H_2_O_2_ for 4–6 h). A uniform scratch was created using a sterile pipette tip. After washing, fresh medium containing the test samples was added. Cell migration was monitored at 0, 24, and 48 h. Wound areas were quantified using ImageJ to calculate closure rates. For proliferation assessment, cells were fixed, permeabilized, stained for F-actin (Actin-Tracker Green) and nuclei (DAPI), and imaged.

### VEGF secretion assay

Cells were pretreated with the HG/OS model as described for live/dead staining. The medium was then replaced with fresh medium containing the test samples. After 48 h of incubation, culture supernatants were collected, and VEGF levels were quantified using a commercial ELISA kit.

### Western blot analysis

After treatment, cells were lysed on ice with RIPA buffer containing protease inhibitors. Lysates were centrifuged, and the supernatant was collected. Total protein concentration was determined using a BCA assay. Equal amounts of protein were separated by SDS-PAGE, transferred to PVDF membranes, and probed with specific primary antibodies overnight at 4 °C, followed by incubation with appropriate HRP-conjugated secondary antibodies. Protein bands were visualized using an enhanced chemiluminescence (ECL) detection system.

### Co-immunoprecipitation (Co-IP) assay for SIRT1 ubiquitination

Prior to lysis, the proteasome inhibitor MG132 (10 µM) was added to the culture medium for 4–6 h. Equal amounts of total protein lysate were pre-cleared and then incubated with an anti-SIRT1 antibody at room temperature for 2 h. Protein A/G magnetic beads were added, and the mixture was incubated at 4 °C for 2 h. The beads were washed thoroughly, and bound proteins were eluted by boiling in 2× Laemmli buffer. The eluates were analyzed by Western blot using an anti-ubiquitin antibody to detect SIRT1 ubiquitination levels.

### Diabetic animal wound model

All animal procedures were approved by the Institutional Animal Care and Use Committee (IACUC) of Tongji Medical College, Huazhong University of Science and Technology (protocol number: 2789[2021]). Male Sprague-Dawley rats (8–10 weeks old) were acclimatized for one week. Type 2 diabetes was induced by a single intraperitoneal injection of streptozotocin (60 mg/kg). Rats with non-fasting blood glucose levels consistently > 15 mM were considered diabetic. Under anesthesia, a full-thickness circular wound (1.5 cm diameter) was created on the shaved dorsal skin. Rats were randomly assigned to experimental groups.

### Hemolysis assay

Fresh anticoagulated blood was collected. Red blood cells (RBCs) were isolated, washed, and resuspended in PBS to a 5% (v/v) suspension. Sec and MOF-818@Sec were dispersed in PBS at concentrations of 0–200 µg/mL. Each solution (600 µL) was mixed with 150 µL of the RBC suspension (*n* = 3), incubated at 37 °C for 1 h, and centrifuged. Hemolysis was assessed by measuring the absorbance of the supernatant at 540 nm. PBS and deionized water served as negative and positive controls, respectively. Hemolysis ratio (%) was calculated as:

$$\begin{aligned}&\:\mathrm{H}\mathrm{e}\mathrm{m}\mathrm{o}\mathrm{l}\mathrm{y}\mathrm{s}\mathrm{i}\mathrm{s}\:\mathrm{R}\mathrm{a}\mathrm{t}\mathrm{i}\mathrm{o}\:\left({\%}\right)\:\\& \quad=\:\mathrm{(}{\mathrm{OD}}_{\mathrm{t}}\mathrm{-}{\mathrm{OD}}_{\mathrm{p}})/\left({\mathrm{OD}}_{\mathrm{w}}\mathrm{-}{\mathrm{OD}}_{\mathrm{p}}\right)\times{100\%}\end{aligned}$$

where OD_t_, OD_p_, and OD_w_ denote the absorbance of the test, negative control (PBS), and positive control groups, respectively.

### In vitro coagulation effect and blood clotting index (BCI)

For the coagulation assay, fresh citrated rat blood was mixed with sample solutions (Sec, MOF-818, MOF-818@Sec, or P-G^MOF−818@Sec^ at 400 µg/mL) and CaCl_2_ (0.1 M) to initiate clotting. The mixture was gently shaken, and aliquots were transferred to a 96-well plate at various time points. PBS was added to remove non-coagulated blood, and clot formation was visually recorded. The coagulation time was defined as the earliest time point when a stable clot remained. For BCI determination, sample fragments were incubated with whole blood and CaCl_2_ at 37 °C for 2 min. Deionized water was added to lyse unclotted RBCs. After centrifugation, supernatant absorbance was measured at 540 nm. The BCI was calculated as follows:

$$\:\mathrm{B}\mathrm{C}\mathrm{I}\:\left({\%}\right)\:=\:\mathrm{(}{\mathrm{OD}}_{\mathrm{sample}}/{\mathrm{OD}}_{\mathrm{control}}\times{100\%}$$

where OD_Control_ is the absorbance measured with PBS instead of the sample.

### In vivo biodistribution and metal ion analysis

For fluorescence tracing, indocyanine green (ICG)-labeled MOF-818@Sec or ICG-labeled P-G^MOF−818@Sec^ was applied topically. Fluorescence signals at the wound site and in major organs (heart, liver, spleen, lung, kidney) were monitored at 0, 1, 2, 4, 6, 12, 24, and 48 h post-application using a small-animal in vivo imaging system.

For quantitative analysis of MOF-derived metal biodistribution, Cu and Zr contents in major organs and wound skin were measured by inductively coupled plasma mass spectrometry (ICP-MS) at 3, 6, 12, 24, and 48 h post-treatment. Tissues were digested with concentrated nitric acid, diluted, and analyzed. Metal contents were normalized to tissue wet weight (µg/g tissue).

### In vivo wound healing evaluation

Wounds were photographed on days 0, 3, 6, 9, 12, 15, and 18, and the wound closure rate was calculated. For in vivo antibacterial assessment, wound exudates were collected with sterile swabs on days 3 and 9, resuspended in saline, serially diluted, plated on agar, and incubated. CFUs were counted to quantify bacterial load.

### Histological and molecular analysis of wound tissues

On day 18, wound tissues were harvested. One portion was fixed, paraffin-embedded, and sectioned for hematoxylin and eosin (H&E) staining, Masson’s trichrome staining, and immunofluorescence staining for CD86, CD206, and CD31. Another portion was homogenized. The supernatants were used to quantify VEGF, interleukin-10 (IL-10), tumor necrosis factor-α (TNF-α), and interleukin-6 (IL-6) levels via ELISA. Protein expression of SIRT1/FOXO3a pathway components was assessed by Western blot.

### Serum biochemical analysis

Blood samples were collected at the endpoint. Serum was separated by centrifugation. Liver function markers alanine aminotransferase (ALT) and aspartate aminotransferase (AST), and renal function markers serum urea (UREA) and creatinine (CREA) were measured using commercial assay kits.

### Statistical analysis

Data are presented as mean ± standard deviation (SD) from at least three independent experiments. Statistical significance was determined using Student’s t-test for two-group comparisons or one-way analysis of variance (ANOVA) with Tukey’s post-hoc test for multiple comparisons. Normality was verified using the Shapiro-Wilk test. Statistical analyses were performed using GraphPad Prism 7.0. A p-value < 0.05 was considered statistically significant.

## Results

### Structural and functional characterization of MOF-818

**Fig. 2:**
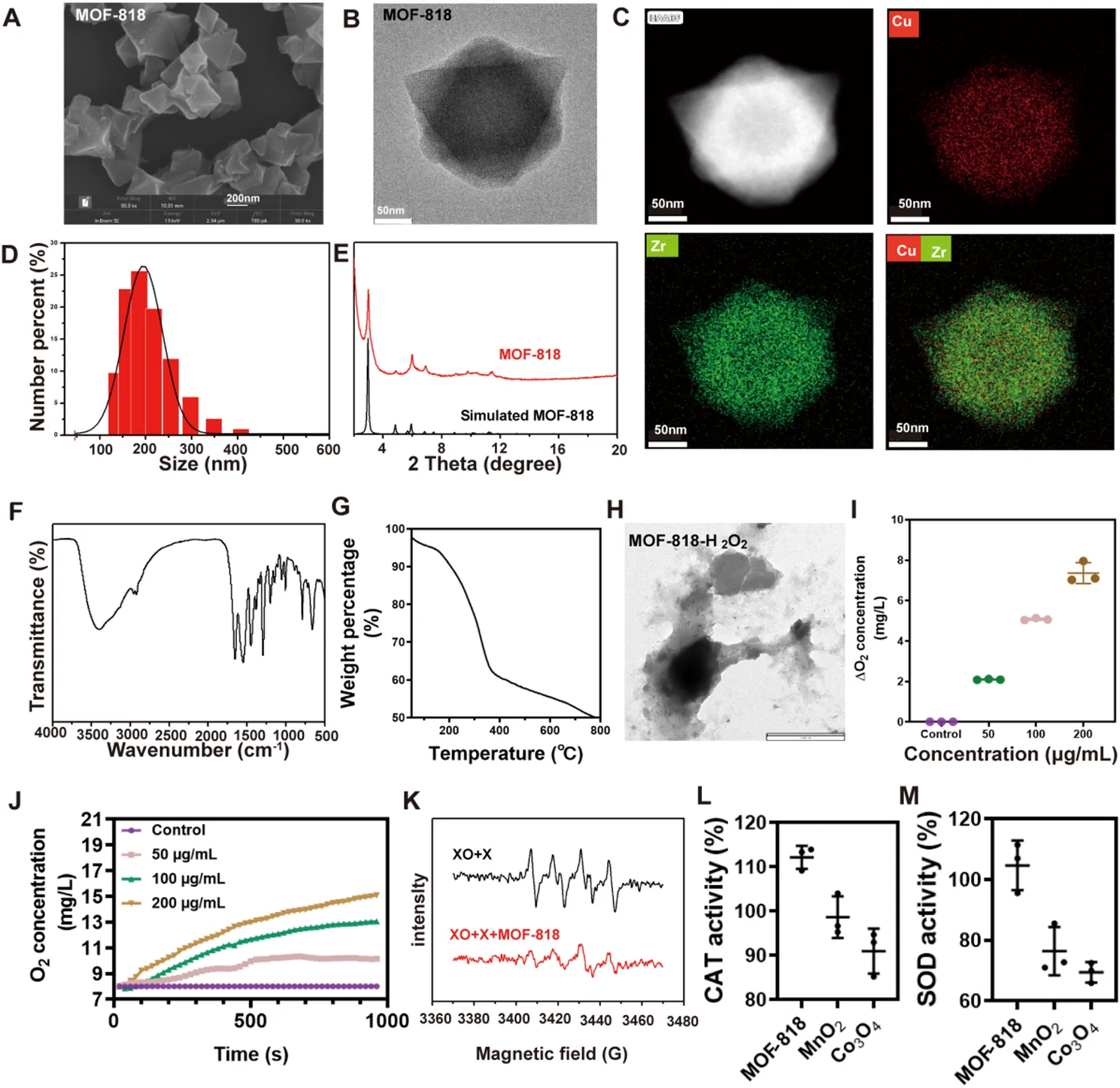
Characterization of MOF-818. (**A**) SEM image of MOF-818. Scale bar = 200 nm. (**B**) TEM image of MOF-818. Scale bar = 50 nm. (**C**) EDS mapping of MOF-818. Scale bar = 50 nm. (**D**) DLS analysis of MOF-818. (**E**) XRD pattern of MOF-818. (**F**) FT-IR spectrum of MOF-818. (**G**) TGA curve of MOF-818. (**H**) TEM image of MOF-818 after treatment with 50 µM H_2_O_2_. (**I**) H_2_O_2_ decomposition kinetics of MOF-818. (**J**) Oxygen generation induced by MOF-818. (**K**) Superoxide anion scavenging performance of MOF-818. (**L**) CAT-like activity and (**M**) SOD-like activity of MOF-818. Data are presented as mean ± SD (*n*=3)

MOF-818 was successfully synthesized as uniform octahedral nanocrystals, as confirmed by SEM and TEM (Fig. 2A, B). Energy-dispersive X-ray spectroscopy (EDS) mapping confirmed the homogeneous distribution of Zr and Cu elements (Fig. 2C). Dynamic light scattering (DLS) indicated a hydrodynamic diameter of 206.3 nm (Fig. 2D). Powder X-ray diffraction (XRD) patterns matched the simulated spectrum, confirming high crystallinity and phase purity (Fig. 2E). Fourier-transform infrared (FT-IR) spectroscopy verified successful coordination bonding (Fig. 2F). Nitrogen adsorption-desorption isotherms revealed a high Brunauer–Emmett–Teller (BET) surface area of 1034.33 m^2^/g and a mesoporous structure (Figure S1). Thermogravimetric analysis (TGA) indicated stability up to 160 °C (Fig. 2G). MOF-818 exhibited ROS-responsive degradation upon exposure to 50 µM H_2_O_2_ (Fig. 2H). Catalytic assays demonstrated efficient H_2_O_2_ decomposition (Fig. 2I, J) and potent superoxide anion (·O_2_^–^) scavenging activity, as evidenced by electron paramagnetic resonance (EPR) spectroscopy (Fig. 2K). Comparative studies confirmed that MOF-818 outperformed conventional nanozymes (MnO_2_, Co_3_O_4_) in both catalase (CAT)- and superoxide dismutase (SOD)-mimetic activities (Fig. 2L, M), highlighting its dual nanozyme functionality.

### Characterization of P-G^MOF-818@Sec^

**Fig. 3:**
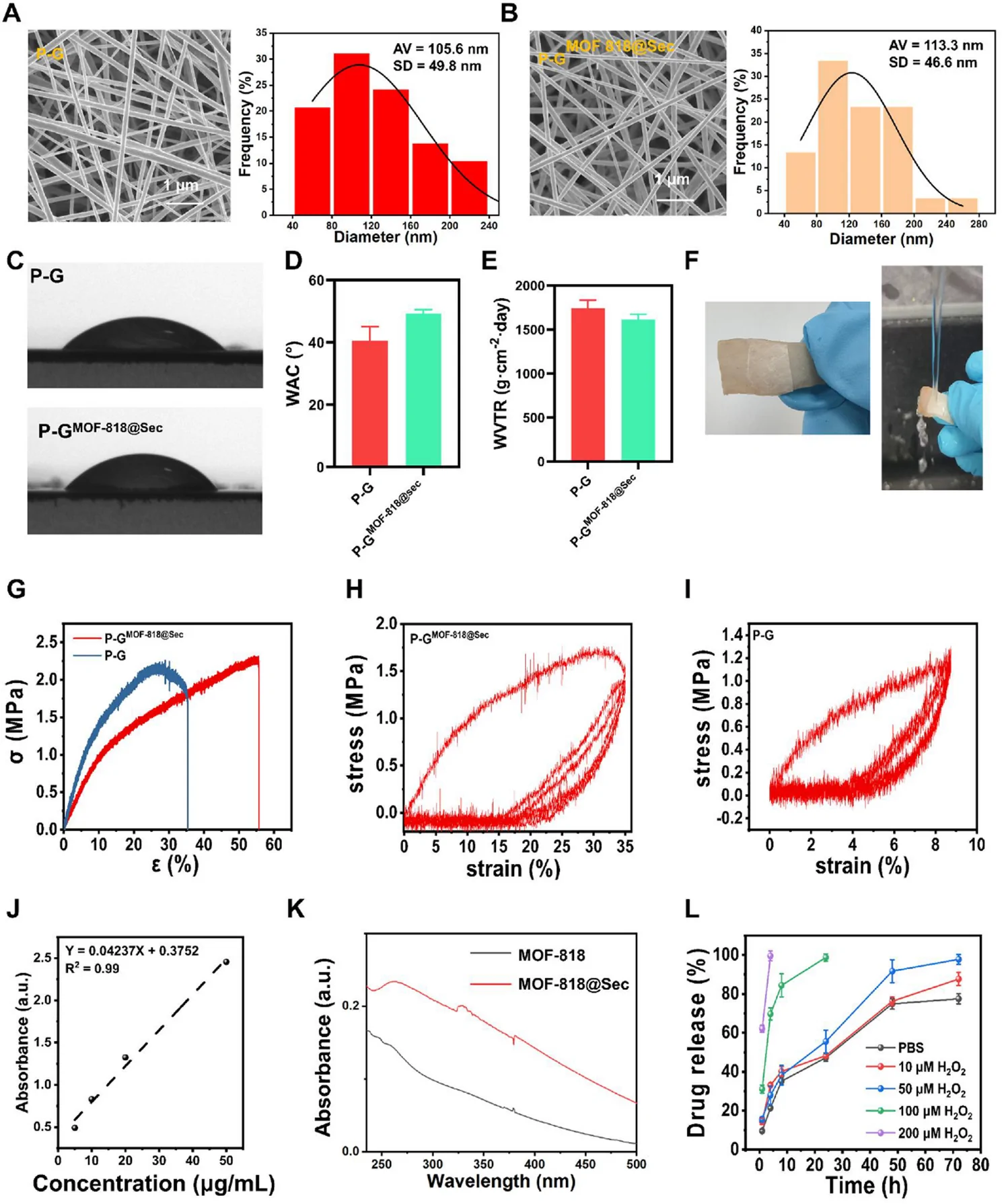
Physicochemical characterization, mechanical properties, and ROS-responsive Sec release behavior of P-G^MOF-818@Sec^. **(A**) Morphology and diameter distribution of P-G. Scale bar = 1 μm. (**B**) Morphology and diameter distribution of P-G^MOF-818@Sec^. Scale bar = 1 μm. (**C–D**) Water contact angle analysis of P-G and P-G^MOF-818@Sec^ membranes. (**E**) Water vapor transmission rate (WVTR) of P-G and P-G^MOF-818@Sec^ membranes. (**F**) Adhesive strength of the membrane to skin tissue. (**G**) Representative tensile stress–strain curves of P-G and P-G^MOF-818@Sec^ membranes. (**H**) Cyclic tensile behavior of P-G^MOF-818@Sec^ membranes at a maximum strain of 35%. (**I**) Cyclic tensile behavior of P-G membranes at a maximum strain of 10%. (**J**) Standard curve of Sec. (**K**) Drug loading capacity (DLC) and encapsulation efficiency (EE) of P-G^MOF-818@Sec^. (**L**) In vitro Sec release profiles from P-G^MOF-818@Sec^ under different H_2_O_2_ concentrations. Data are presented as mean ± SD (*n*=3)

MOF-818@Sec was successfully incorporated into the electrospun P-G matrix, yielding the composite membrane P-G^MOF-818@Sec^. SEM imaging revealed an interwoven nanofibrous morphology. The average fiber diameter increased slightly from ~ 105.6 nm for pristine P-G to ~ 113 nm for P-G^MOF-818@Sec^, with MOF-818@Sec particles uniformly dispersed within the fibers (Fig. 3A, B). The composite membrane retained properties suitable for wound dressing: it maintained moderate hydrophilicity (water contact angle increased from 43.4° to 48.5°) and an appropriate water vapor transmission rate (decreased from 1744 to 1612 g/m^2^/day) (Fig. 3C–E). The membrane also exhibited strong adhesion to skin, attributed to hydrogen bonding from gelatin (Fig. 3F). Mechanical characterization showed that P-G^MOF-818@Sec^ maintained a tensile strength of 2.3 MPa, similar to P-G, while its Young’s modulus increased to 18.1 MPa and elongation at break decreased to 29.0% (Fig. 3G, Table S1). Cyclic tensile tests confirmed that both membranes maintained structural integrity after three loading-unloading cycles (Fig. 3H, I). Based on a Sec standard curve (Fig. 3J), the drug loading capacity and encapsulation efficiency were determined to be 30.1% and 44%, respectively (Fig. 3K). The release of Sec from P-G^MOF-818@Sec^ was progressively accelerated with increasing H_2_O_2_ concentration (0, 10, 50, 100, 200 µM), confirming ROS-responsive behavior (Fig. 3L). Zero-order kinetic fitting revealed that the apparent release rate constant (k) increased from 5.35 (0 µM H_2_O_2_) to 24.86 (200 µM H_2_O_2_) (Table S2), demonstrating a positive correlation between release rate and oxidative stimulus.

### Antibacterial properties and cytocompatibility of P-G^MOF−818@Sec^

**Fig. 4:**
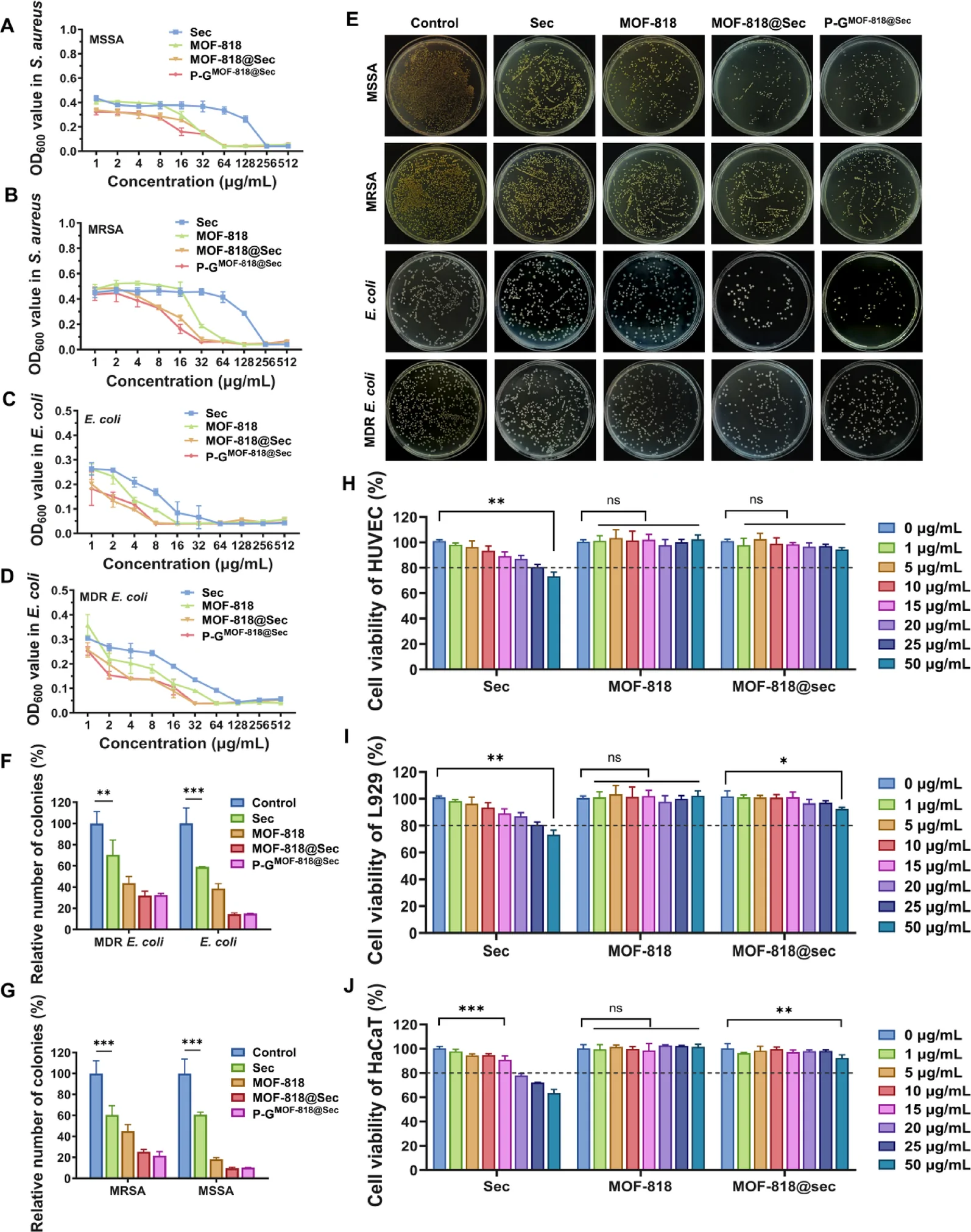
Evaluation of antibacterial properties and cytocompatibility of P-G^MOF-818@Sec^. (**A–D**) OD_600_-based MIC curves of Sec, MOF-818, MOF-818@Sec, and P-G^MOF-818@Sec^ against MSSA, MRSA, *E. coli*, and MDR *E. coli*. (**E**) Representative plate count images of MSSA, MRSA, *E. coli*, and MDR *E. coli* after different treatments. (**F**) Quantification of CFUs for MSSA and MRSA. (**G**) Quantification of CFUs for *E. coli* and MDR *E. coli*. (**H–J**) Cell viability of HUVECs, L929, and HaCaT cells after different treatments, as determined by CCK-8 assay. Data are presented as mean ± SD (*n*=3). *p < 0.05, **p < 0.01, and ***p < 0.001

The antibacterial activity was evaluated against antibiotic-sensitive and drug-resistant strains. Minimum inhibitory concentration (MIC) assays showed that free Sec had no direct antibacterial effect, whereas MOF-818 exhibited intrinsic activity with MICs of 64, 128, 32, and 64 µg/mL against MSSA, MRSA, *E. coli*, and MDR *E. coli*, respectively (Fig. 4A–D). The activity was retained in MOF-818@Sec and P-GMOF-818@Sec. Colony counting and Live/Dead staining confirmed that MOF-818@Sec and P-GMOF-818@Sec significantly reduced viable bacterial counts of all tested strains, with P-GMOF-818@Sec showing the strongest effect (Fig. 4E–G, Figure S2). Cytocompatibility was assessed in HUVECs, L929 fibroblasts, and HaCaT keratinocytes. MOF-818 and MOF-818@Sec exhibited excellent biocompatibility, maintaining cell viability > 90% at concentrations up to 50 µg/mL. In contrast, free Sec showed concentration-dependent cytotoxicity, which was mitigated upon loading into MOF-818 (Fig. 4H–J).

### P-G^MOF−818@Sec^ alleviates oxidative stress

**Fig. 5:**
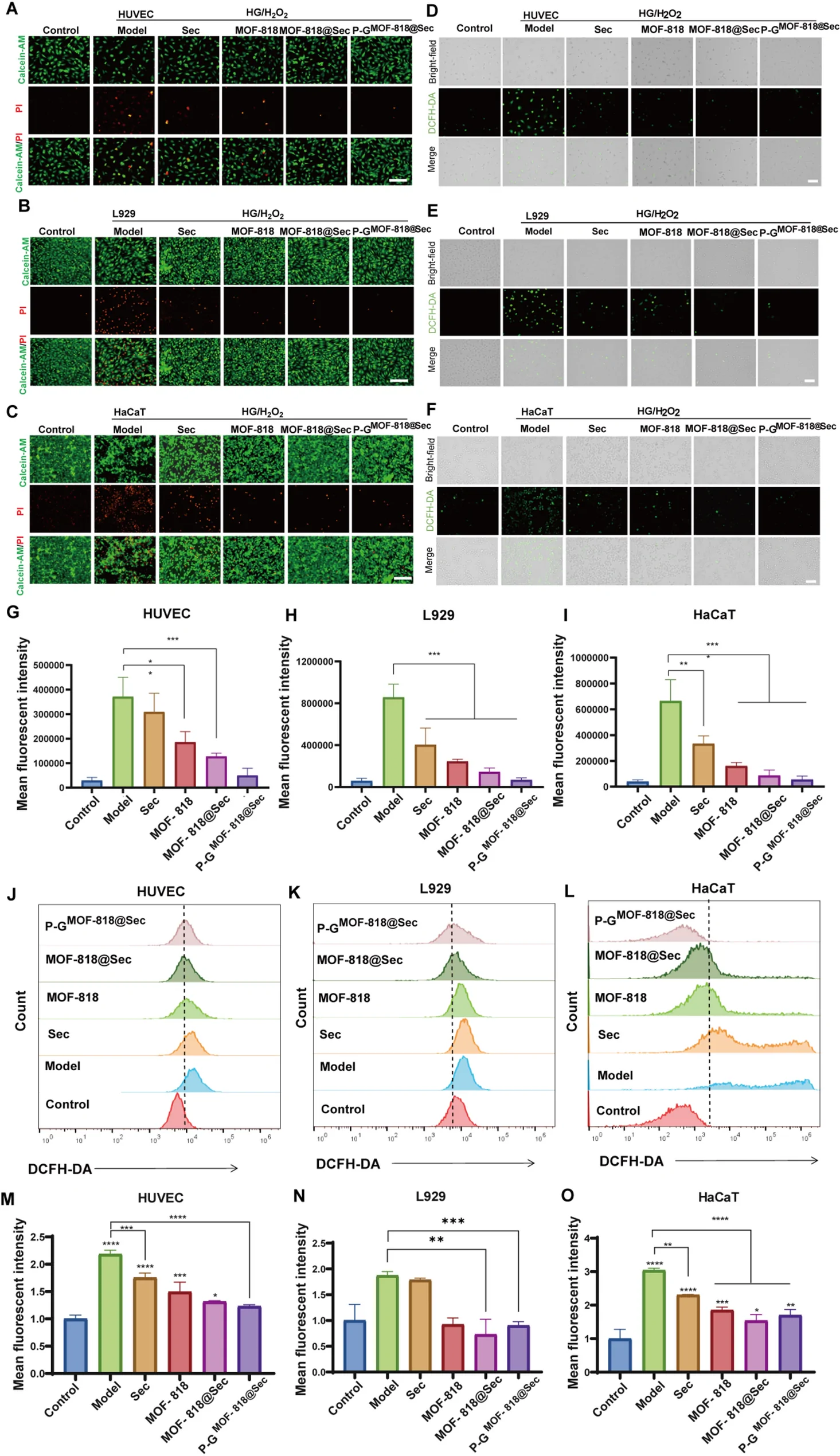
Assessment of intracellular ROS levels and cell viability. (**A–C**) Representative live/dead staining images of HUVECs, L929, and HaCaT cells. Scale bar = 125 μm. (**D–F**) Representative fluorescence images of HUVECs, L929 cells, and HaCaT cells stained with DCFH-DA for ROS detection. Scale bar = 125 μm. (**G–I**) Quantification of DCFH-DA fluorescence indicating ROS levels in HUVECs, L929, and HaCaT cells. (**J–L**) Flow cytometric analysis of ROS levels in HUVECs, L929, and HaCaT cells. (**M–O**) Quantification of flow cytometry data in HUVECs, L929, and HaCaT cells. Data are presented as mean ± SD (*n* = 3). *p < 0.05, **p < 0.01, and ***p < 0.001

To mimic the hyperglycemic and oxidative stress microenvironment of diabetic chronic wounds, a model was established using 80 mM glucose and 300 µM H_2_O_2_ (Figures S3, S4). The cytoprotective effect of P-G^MOF−818@Sec^ under high-glucose/oxidative stress was assessed by Live/Dead staining (Fig. 5A–C). According to statistical results, all treatment groups showed improved cell viability compared to the PBS-treated model group. MOF-818@Sec outperformed MOF-818 or Sec alone, underscoring the synergistic benefit of combining Sec with the MOF-818 nanocarrier. Notably, P-G^MOF−818@Sec^ exhibited the highest cell viability, which may be attributed to the bioactive gelatin-containing P-G matrix, efficient Sec delivery, and activation of the SIRT1/FOXO3a signaling pathway, a key regulator of antioxidant defense and cell survival (Figure S5). These results suggest a synergistic cytoprotective effect arising from the integration of Sec-loaded MOF-818 with the P-G nanofibrous membrane.

Intracellular ROS levels were assessed via DCFH-DA fluorescence (Fig. 5D–F): L929 cells under high glucose/H_2_O_2_ stress showed markedly increased green fluorescence versus control, confirming successful modeling. Treatment with Sec or MOF-818 alone reduced ROS, but MOF-818@Sec exhibited superior suppression, indicating synergistic antioxidant effects. Encapsulation in the P-G membrane (P-G^MOF−818@Sec^) preserved this activity (Fig. 5G). Similar results were observed in HUVECs and HaCaT cells (Fig. 5H, I), demonstrating broad efficacy across key wound-healing cell types. Flow cytometry quantification (Fig. 5J–L) corroborated these findings: all treatment groups showed lower ROS than the model group, with MOF-818@Sec and P-G^MOF−818@Sec^ displaying the strongest effects (Fig. 5M–O). Collectively, the data confirm that P-G^MOF−818@Sec^ effectively mitigates oxidative stress without compromising bioactivity upon integration into the fibrous membrane.

### P-G^MOF−818@Sec^ promotes cell migration, proliferation and angiogenesis

**Fig. 6:**
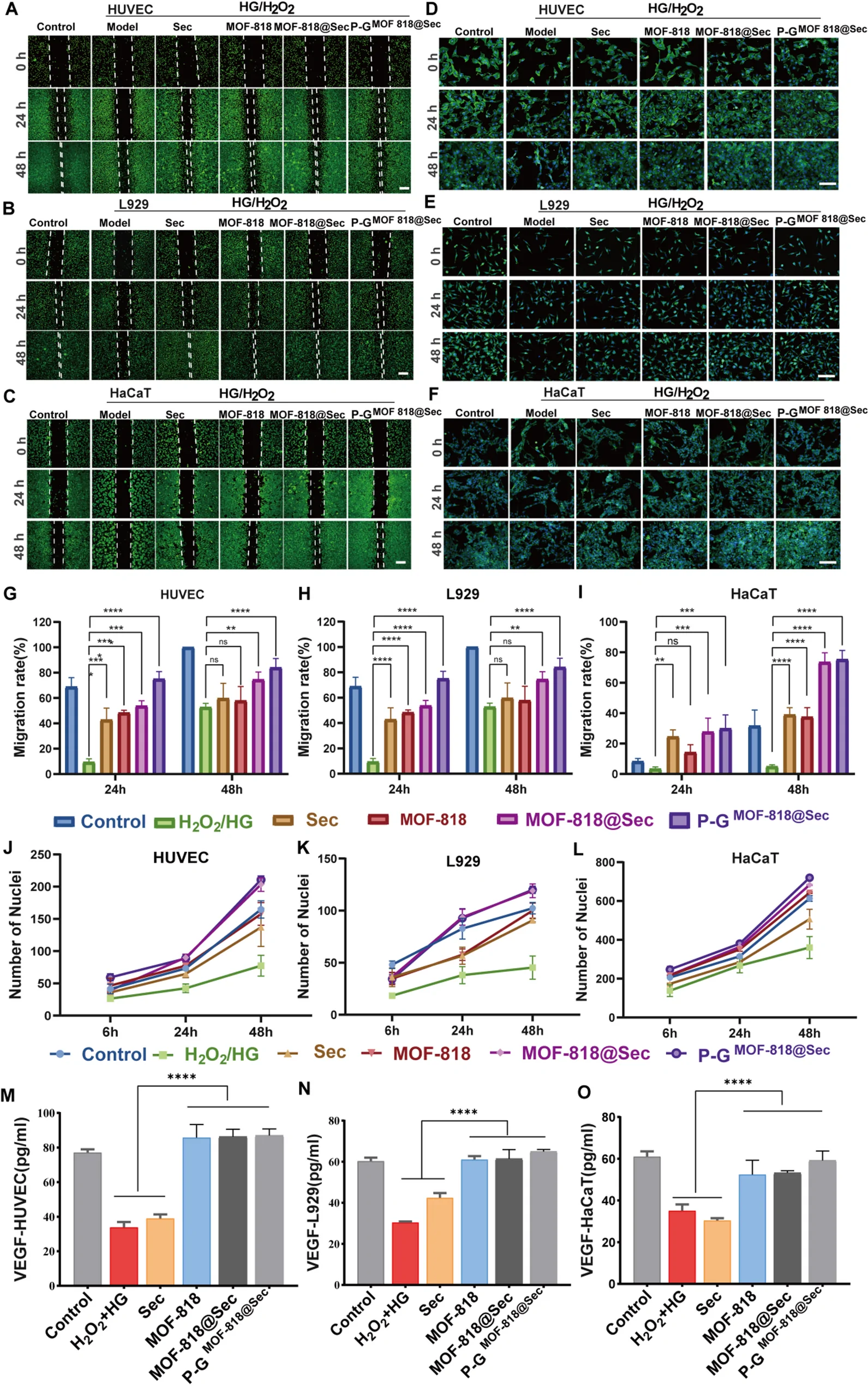
P-G^MOF−818@Sec^ promotes cell migration, proliferation, and angiogenesis-related VEGF secretion. (**A–C**) Representative images of migration assays for HUVECs, L929, and HaCaT cells. Scale bar = 100 μm. (**D–F**) Representative fluorescence images showing cell proliferation and cytoskeletal organization in HUVECs, L929, and HaCaT cells. Scale bar = 100 μm. (**G–I**) Quantitative analysis of the corresponding migration rates. (**J–L**) Quantitative analysis of the corresponding proliferation rates. (**M–O**) VEGF secretion levels in the culture supernatants of HUVECs, L929, and HaCaT cells quantified by enzyme-linked immunosorbent assay (ELISA). Data are presented as mean ± SD (*n* = 3). *p < 0.05, **p < 0.01, and ***p < 0.001

The impact on cellular functions critical for wound healing was evaluated under HG/OS conditions. Scratch wound assays revealed that HG/OS impaired the migration of HUVECs, L929, and HaCaT cells. All treatments partially restored migration, with P-G^MOF−818@Sec^ demonstrating the most potent pro-migratory effect (Fig. 6A–C, G–I). Phalloidin staining for cytoskeletal organization indicated that P-G^MOF−818@Sec^ most effectively reversed the HG/OS-induced suppression of cell proliferation (Fig. 6D–F, J–L). Furthermore, ELISA analysis of culture supernatants showed that P-G^MOF−818@Sec^ treatment induced the highest secretion of vascular endothelial growth factor (VEGF), a key angiogenic regulator, in all three cell types (Fig. 6M–O).

### P-G^MOF−818@Sec^ inhibits SIRT1 ubiquitination

**Fig. 7:**
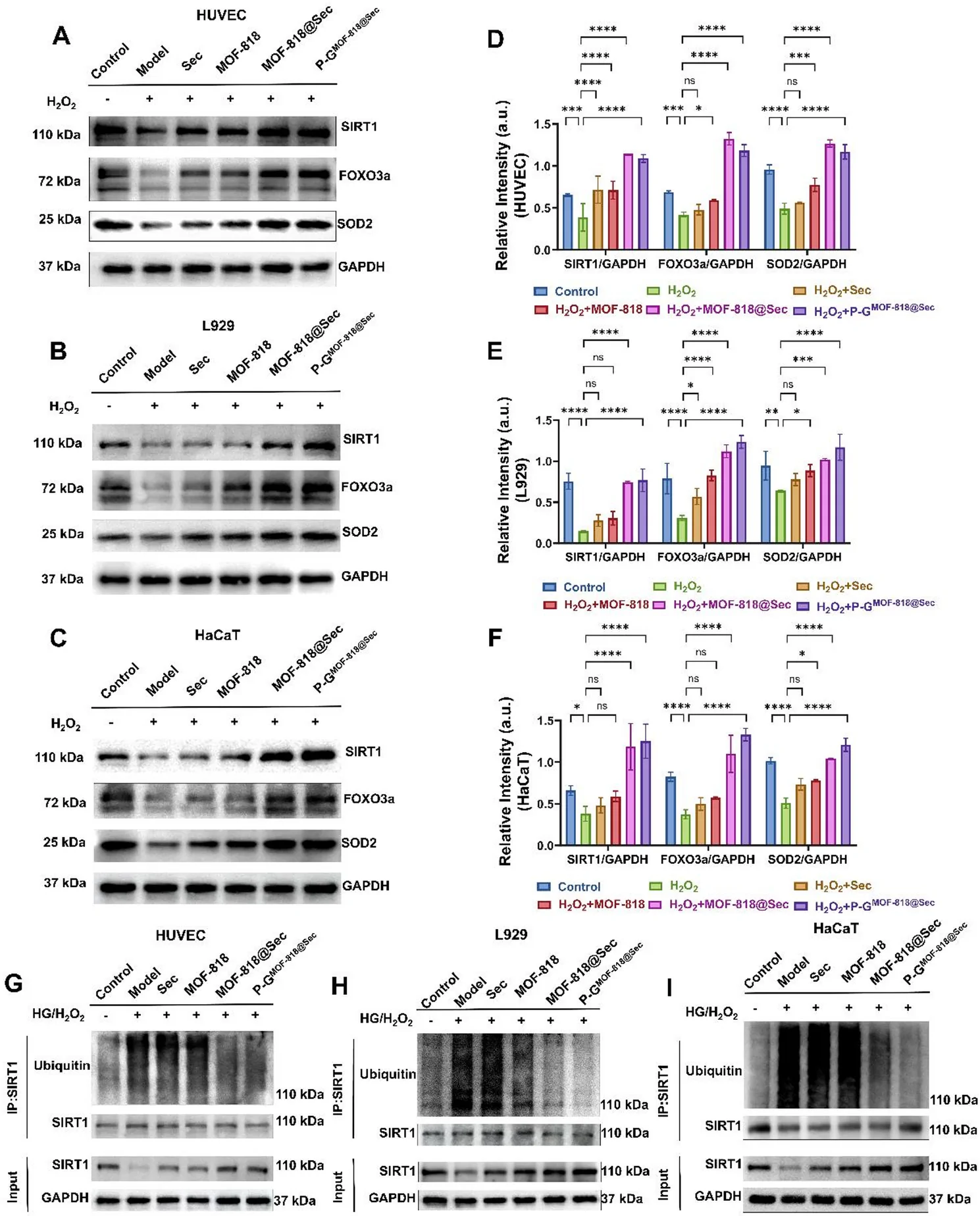
P-G^MOF−818@Sec^ restores SIRT1/FOXO3a/SOD2 signaling and suppresses SIRT1 ubiquitination. (**A–C**) Western blot bands of SIRT1/FOXO3a/SOD2 pathway-related proteins in HUVECs, L929, and HaCaT cells after different treatments. (**D–F**) Quantitative analysis of the corresponding protein expression levels. (**G–I**) Co-immunoprecipitation analysis of SIRT1 ubiquitination levels in HUVECs, L929, and HaCaT cells after different treatments. Data are presented as mean ± SD (*n* = 3). *p < 0.05, **p < 0.01, and ***p < 0.001

We investigated the molecular mechanism underlying the observed benefits, focusing on the SIRT1/FOXO3a/SOD2 axis. Western blot analysis showed that HG/OS downregulated the expression of SIRT1, FOXO3a, and SOD2. Treatment with MOF-818@Sec and P-G^MOF−818@Sec^ effectively restored the expression levels of these proteins (Fig. 7A–F). Co-immunoprecipitation assays revealed that HG/OS induced significant ubiquitination of SIRT1, which was markedly suppressed by treatment with MOF-818@Sec and P-G^MOF−818@Sec^ (Fig. 7G–I). This confirms that Sec, released from the platform, acts as a Fyn kinase inhibitor to block Fyn-mediated ubiquitination of SIRT1, thereby preventing its proteasomal degradation and stabilizing the protein to activate its downstream antioxidant and pro-survival signaling.

### In vivo evaluation of diabetic wound healing

**Fig. 8:**
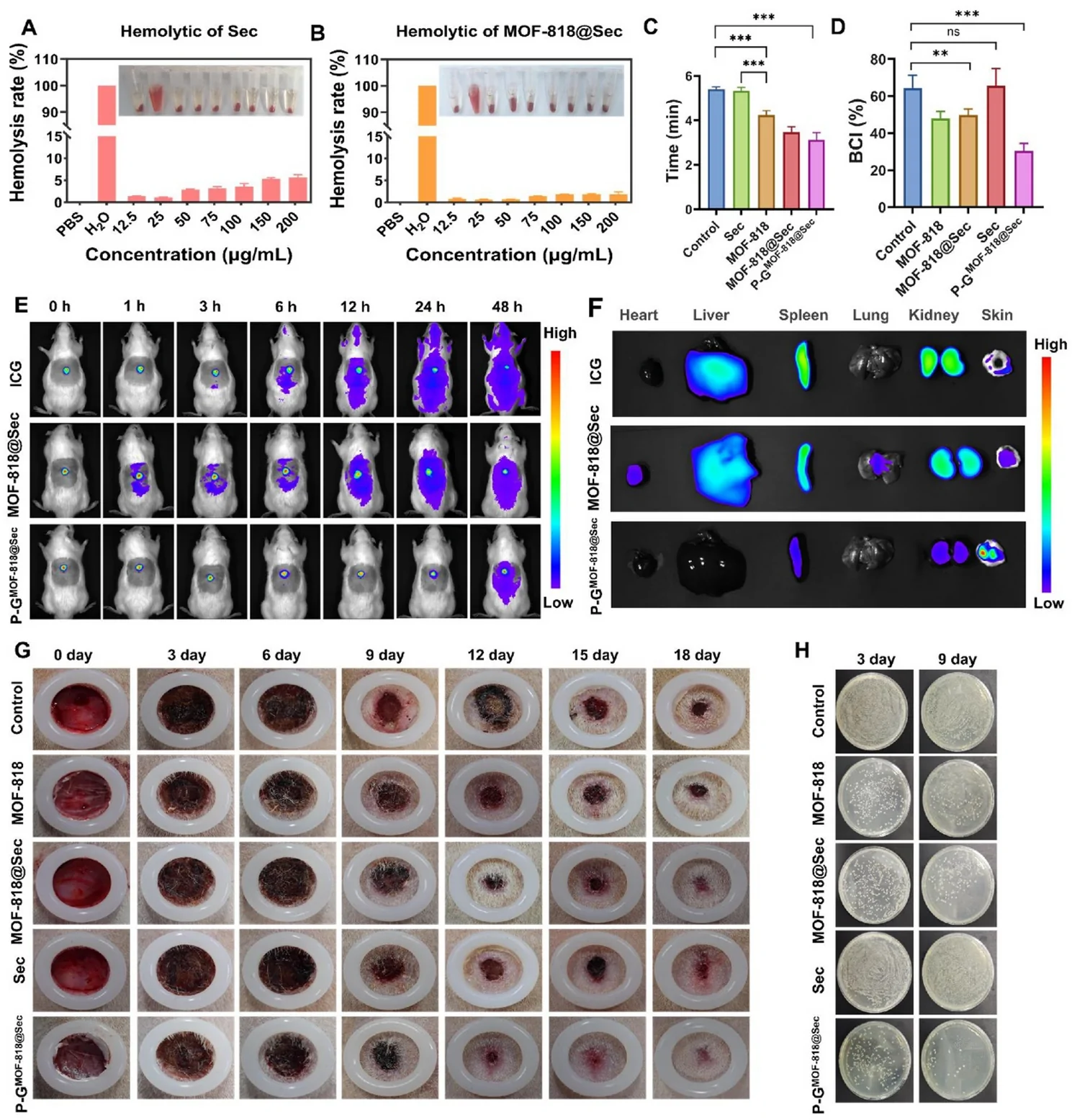
In vivo therapeutic efficacy, blood compatibility, and local retention of P-G^MOF−818@Sec^. (**A**) Hemolysis rates of Sec at different concentrations. (**B**) Hemolysis rates of MOF-818@Sec at different concentrations. (**C**) Coagulation time and (**D**) Blood clotting index (BCI) of different treatments. (**E**) In vivo fluorescence retention of free ICG, ICG-loaded MOF-818@Sec, and ICG-loaded P-G^MOF−818@Sec^ at the wound site over time. (**F**) Ex vivo biodistribution of ICG fluorescence in major organs and wound tissues. (**G**) Representative photographs showing wound healing progression under different treatments. (**H**) In vivo antibacterial activity at wound sites on days 3 and 9. Data are presented as mean ± SD (*n* = 3). *p < 0.05, **p < 0.01, and ***p < 0.001

Prior to in vivo studies, hemolysis assays confirmed the excellent blood compatibility of Sec and MOF-818@Sec (Fig. 8A, B). In vitro coagulation tests and blood clotting index (BCI) analysis demonstrated that P-G^MOF−818@Sec^ promoted clot formation, indicating hemostatic potential (Fig. 8C, D, Figure S6). A diabetic rat full-thickness wound model was established (Figure S7). In vivo fluorescence imaging using ICG-labeled materials showed that the P-G matrix significantly enhanced the local retention of MOF-818@Sec at the wound site over 48 h and reduced its systemic distribution to major organs compared to free ICG or ICG-MOF-818@Sec (Fig. 8E, F). Inductively coupled plasma mass spectrometry (ICP-MS) analysis quantitatively confirmed that P-G^MOF−818@Sec^ led to higher retention of Cu and Zr ions at the wound site and lower accumulation in vital organs (heart, liver, spleen, lung, kidney) within 48 h, compared to MOF-818@Sec alone (Figure S8).

In the wound healing study, P-G^MOF−818@Sec^ treatment resulted in the most rapid and complete wound closure compared to all other groups (Fig. 8G). Furthermore, bacterial loads in wound exudates on days 3 and 9 were significantly lower in groups treated with MOF-818, MOF-818@Sec, and P-G^MOF−818@Sec^, confirming in vivo antibacterial efficacy (Fig. 8H).

### P-G^MOF−818@Sec^ promotes healing via the SIRT1 pathway, modulating inflammation and angiogenesis

**Fig. 9:**
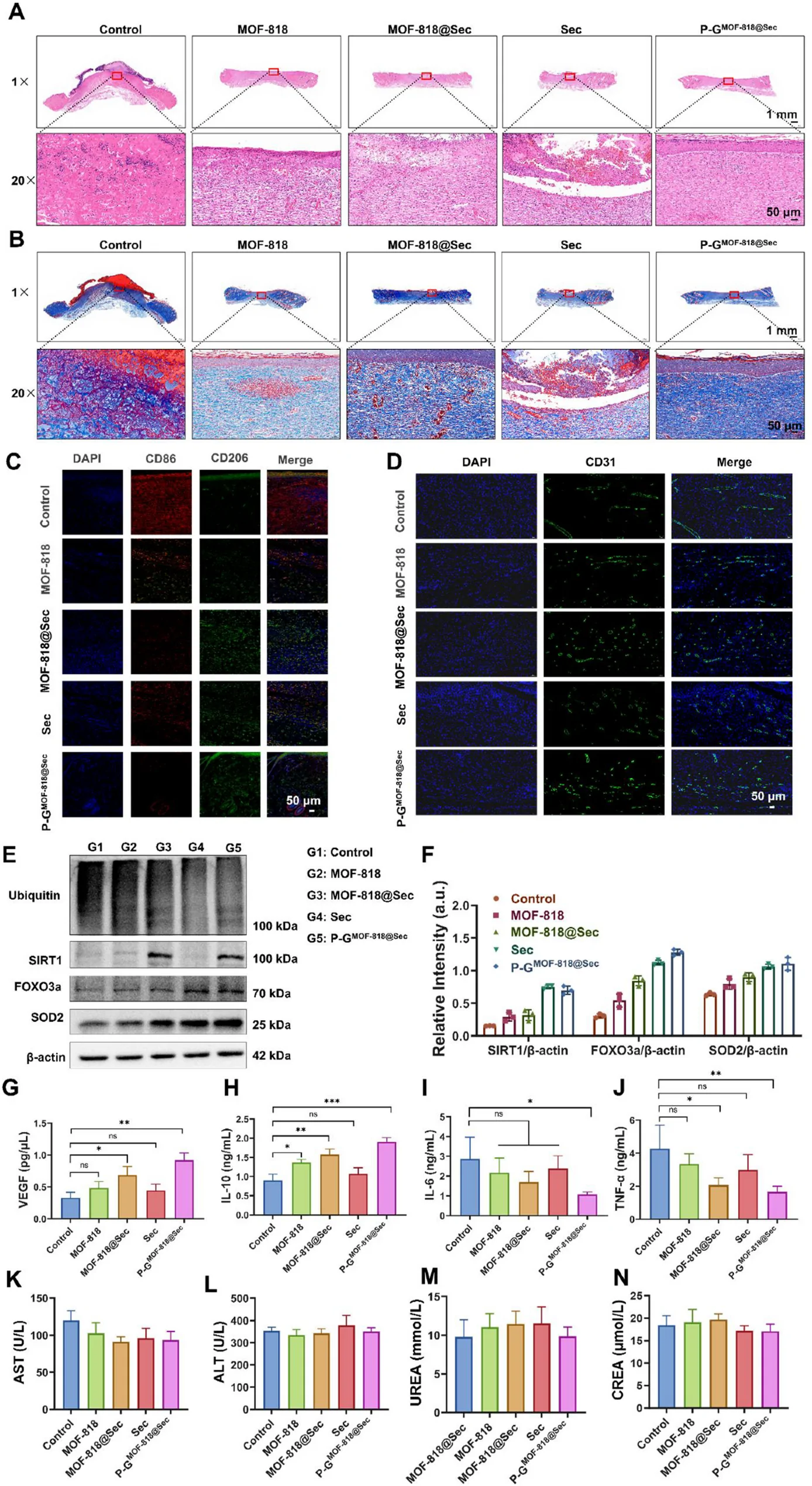
Histological, immunological, biochemical, and molecular analyses of wound tissues after treatment. (**A**) H&E staining of wound sections after different treatments. Scale bars: 1 mm (1×) and 50 μm (20×). (**B**) Masson’s trichrome staining of wound sections after different treatments. Scale bars: 1 mm (1×) and 50 μm (20×). (**C**) Immunofluorescence staining of macrophage polarization markers CD86 and CD206. Scale bar = 50 μm. (**D**) Immunofluorescence staining of CD31. Scale bar = 50 μm. (**E**) Western blot analysis of SIRT1 ubiquitination-related protein expression in wound tissues. (**F**) Quantitative analysis of the corresponding protein expression or ubiquitination levels. (**G–J**) Expression levels of VEGF, IL-10, IL-6, and TNF-α in wound tissues. (**K**,** L**) Serum ALT and AST levels after treatment. (**M**,** N**) Serum UREA and CREA levels after treatment. Data are presented as mean ± SD (*n* = 3). *p < 0.05, **p < 0.01, and ***p < 0.001

Histological and molecular analyses of wound tissues on day 18 provided insights into the healing mechanism. H&E and Masson’s trichrome staining revealed that P-G^MOF−818@Sec^-treated wounds exhibited the most mature granulation tissue, reduced inflammatory infiltration, and enhanced collagen deposition (Fig. 9A, B). Immunofluorescence staining showed that P-G^MOF−818@Sec^ treatment effectively promoted a shift in macrophage polarization from pro-inflammatory CD86⁺ (M1) to pro-healing CD206^+^ (M2) phenotypes (Fig. 9C) and significantly increased the density of CD31^+^ blood vessels, indicating robust angiogenesis (Fig. 9D). Western blot analysis of wound tissue homogenates confirmed that P-G^MOF−818@Sec^ treatment reduced SIRT1 ubiquitination and increased SOD2 expression (Fig. 9E, F). ELISA of wound homogenates revealed that P-G^MOF−818@Sec^ upregulated the levels of pro-angiogenic VEGF and anti-inflammatory IL-10, while downregulating pro-inflammatory cytokines IL-6 and TNF-α (Fig. 9G–J). Serum biochemical analysis indicated no significant abnormalities in liver (ALT, AST) or kidney (UREA, CREA) function markers after treatment, supporting preliminary in vivo biosafety (Fig. 9K–N).

## Discussion

This study demonstrates that the P-G^MOF−818@Sec^ nanofibrous platform effectively promotes diabetic wound healing through coordinated regulation of oxidative stress, bacterial infection, inflammation, angiogenesis, and tissue remodeling. The electrospun P-G membrane serves as a wound-conformal scaffold, enabling sustained local delivery—a well-established advantage of nanofibrous dressings [17, 24–32]. MOF-818 contributes ROS-responsive degradation, intrinsic dual nanozyme activities (SOD and CAT), and antibacterial properties, aligning with previous reports on MOF-based therapeutic systems [33–40]. The small molecule saracatinib (Sec) functions by inhibiting Fyn kinase, thereby blocking Fyn-mediated ubiquitination and degradation of SIRT1. This stabilizes SIRT1 and activates the downstream SIRT1/FOXO3a/SOD2 antioxidant pathway, bolstering endogenous cellular defense mechanisms [11–14, 41]. This intracellular pathway activation complements the extracellular ROS-scavenging action of MOF-818, creating a synergistic effect that alleviates oxidative damage in key wound cells and supports their migratory, proliferative, and pro-angiogenic functions.

A distinguishing feature of P-G^MOF−818@Sec^ is its integrated “extracellular-intracellular” intervention strategy. Many reported diabetic wound dressings primarily target extracellular pathological factors, such as scavenging ROS, supplying oxygen, exerting antibacterial effects, or providing passive drug release [7–10, 18–23, 42–44]. For example, immunomodulatory hydrogels have been designed to neutralize ROS, improve oxygenation, and resolve inflammation [22], while inherently antibacterial hydrogels focus on suppressing bacterial colonization [23]. Although these approaches are valuable, they often do not directly address the intracellular loss of key cytoprotective regulators like SIRT1, which is exacerbated by diabetic oxidative stress. In contrast, P-G^MOF−818@Sec^ uniquely combines MOF-818-mediated remodeling of the extracellular oxidative and infectious microenvironment with Sec-mediated restoration of intracellular SIRT1 stability. This dual-level strategy enables simultaneous targeting of multiple interconnected pathological barriers, distinguishing it from conventional multifunctional dressings.

The antibacterial and mechanical evaluations further support the translational relevance of P-G^MOF−818@Sec^. The confirmed activity against drug-resistant clinical isolates like MRSA and MDR *E. coli* addresses a critical challenge in managing infected diabetic wounds [10, 23]. The mechanical tests confirm that the composite membrane retains adequate tensile strength and tolerance to cyclic deformation, suggesting its potential applicability on dynamic wound sites such as the foot. Preliminary in vivo safety assessments are encouraging. ICP-MS analysis indicated that P-G^MOF−818@Sec^ primarily retained MOF-derived Cu and Zr ions at the wound site with limited short-term systemic exposure. Serum biochemistry showed no signs of acute hepatotoxicity or nephrotoxicity. Together with favorable hemocompatibility and hemostatic performance, these data support the preliminary biosafety of topical application.

Nevertheless, toward clinical translation, several aspects warrant further investigation. Future studies should evaluate the long-term metabolism and clearance of Cu/Zr ions, the potential systemic effects of chronic saracatinib-mediated Fyn inhibition, the platform’s efficacy in more clinically complex models (e.g., ischemic infected wounds), as well as scalable fabrication processes and long-term storage stability.

## Conclusion

In summary, we have developed a multifunctional, ROS-responsive nanofibrous dressing, P-G^MOF−818@Sec^, for synergistic diabetic wound therapy. The P-G matrix provides wound adhesion, local retention, and mechanical support. MOF-818 enables pathophysiology-guided, ROS-responsive drug release, nanozyme-mimetic ROS scavenging, and intrinsic antibacterial activity. The released saracatinib inhibits Fyn-mediated ubiquitination of SIRT1, stabilizing this key regulator and activating the endogenous SIRT1/FOXO3a/SOD2 antioxidant pathway. Through this coordinated extracellular and intracellular regulation, P-G^MOF−818@Sec^ effectively reduced oxidative stress and bacterial burden, promoted a pro-healing immune microenvironment (M2 macrophage polarization), enhanced angiogenesis and collagen deposition, and accelerated re-epithelialization, leading to significantly improved diabetic wound healing in vivo. Comprehensive evaluations of its antibacterial spectrum, mechanical properties, metal ion biodistribution, and serum biochemistry further support its potential as a promising and pathophysiology-guided advanced therapeutic strategy for chronic diabetic wounds. Future work should further validate its long-term safety, manufacturability, and therapeutic performance in clinically relevant infected diabetic foot wound models.

## Supplementary Information

Below is the link to the electronic supplementary material.


Supplementary Material 1.



Supplementary Material 2.


## Data Availability

No datasets were generated or analysed during the current study.
