# Supplementary material for: A SIRT1-stabilizing nanofibrous platform via suppression of ubiquitination for enhanced diabetic wound healing: synergistic therapy with saracatinib and MOF-818
Source: J Nanobiotechnology. 2026 Jun 16;24:816. doi: 10.1186/s12951-026-04663-0 (PMC13523385; doi:10.1186/s12951-026-04663-0)
Supplement: Supplementary file 1 [file 12951_2026_4663_MOESM2_ESM.docx]

**Supporting Information**

**A SIRT1-Stabilizing Nanofibrous Platform via Suppression of Ubiquitination for Enhanced Diabetic Wound Healing: Synergistic Therapy with Saracatinib and MOF-818**

Pingping Xue^1#^, Zhi Yin^2, 3, 4#^, Yuhang Chen^5^, Anran Zhang^5^,

Chenyu Zhuo^5^, Hai Du^5^, Nuo Xu^5^, Renqun Mao^6*^ and Xiaofan Yang^5,7*^

1. Department of Pharmacy, Tongji Hospital, Tongji Medical College, Huazhong University of Science and Technology, Wuhan 430030, China
2. Department of Dermatology, The first Affiliated Hospital with Nanjing Medical University, Nanjing 210029, China
3. Chongqing Hospital of Jiangsu Province Hospital, Chongqing 401420, China
4. Chongqing Hospital of The first Affiliated Hospital with Nanjing Medical University, Chongqing 401420, China
5. Department of Hand Surgery, Union Hospital, Tongji Medical College, Huazhong University of Science and Technology, Wuhan 430022, China
6. Department of Hand and Foot Surgery, Shenzhen Nanshan People's Hospital, Shenzhen 518052, China
7. Hubei Key Laboratory of Regenerative Medicine and Multi-disciplinary Translational Research (Huazhong University of Science and Technology), Wuhan 430022, China

* To whom correspondence should be addressed:

a) Xiaofan Yang

E-mail: 2017XH0119@hust.edu.cn

b) Renqun Mao

E-mail: wufeng5408@163.com

^#^Pingping Xue and Zhi Yin contributed equally to this work

**Table S1. Mechanical parameters of P-G and P-G^MOF-818@Sec^ membranes.** Tensile strength, Young’s modulus, and elongation at break were calculated from the tensile stress–strain curves.

| **Group** | **Tensile strength (MPa)** | **Young’s modulus (MPa)** | **Elongation at break (%)** |
| --- | --- | --- | --- |
| P-G | 2.3 | 10.268 | 54.357 |
| P-G^MOF-818@Sec^ | 2.3 | 18.082 | 28.977 |

**Table S2. Apparent zero-order release rate constants k of Sec from P-G^MOF-818@Sec^ under different H_2_O_2_ concentrations.**

| **H_2_O_2_ concentration (μM)** | **k(c‧h^-1^)** |
| --- | --- |
| 0 | 5.35415 |
| 10 | 6.976973 |
| 50 | 10.03285 |
| 100 | 17.42317 |
| 200 | 24.86111 |

**Figure S1. N_2_ adsorption–desorption characterization of MOF-818.** (A) N_2_ adsorption–desorption isotherm. (B) Pore size distribution calculated from the N_2_ adsorption–desorption isotherm.

**Figure S2. Live/Dead bacterial staining of antibiotic-sensitive and drug-resistant bacterial strains after different treatments.** (A) Representative Live/Dead staining images of MSSA, MRSA, *E. coli*, and MDR *E. coli* after treatment with Control, Sec, MOF-818, MOF-818@Sec, and P-G^MOF-818@Sec^. Live bacteria are shown in green, and dead bacteria are shown in red. Scale bar = 50 μm. (B–E) Quantification of the relative live bacterial ratio for MSSA, MRSA, *E. coli*, and MDR *E. coli*, respectively, based on green fluorescence intensity. Data are presented as mean ± SD (n = 3). *p < 0.05, **p < 0.01, and ***p < 0.001.

**Figure S3. Glucose concentration for establishing the HG/OS model screened by CCK-8.**

**Figure S4. H_2_O_2_ concentration for establishing the HG/OS model using CCK-8.**

**Figure S5. Live/dead cell staining to assess the viability of HG/OS model cells after different treatments.**

**Figure S6. Representative physical images of the *in vitro* coagulation assay after different treatments.**

**Figure S7. Measurement of body weight and blood glucose in rats after STZ injection.**

**Figure S8. ICP-MS quantification of MOF-derived metal elements after topical treatment.** (A) Cu contents in the heart, liver, spleen, lung, kidney, and wound skin tissues at 3, 6, 12, 24, and 48 h after MOF-818@Sec or P-G^MOF-818@Sec^ treatment. (B) Zr contents in the heart, liver, spleen, lung, kidney, and wound skin tissues at 3, 6, 12, 24, and 48 h after MOF-818@Sec or P-G^MOF-818@Sec^ treatment. Data are presented as mean ± SD (n = 3). *p < 0.05, **p < 0.01, and ***p < 0.001.
