# Supplementary material for: A SIRT1-stabilizing nanofibrous platform via suppression of ubiquitination for enhanced diabetic wound healing: synergistic therapy with saracatinib and MOF-818
Source: J Nanobiotechnology. 2026 Jun 16;24:816. doi: 10.1186/s12951-026-04663-0 (PMC13523385; doi:10.1186/s12951-026-04663-0)
Supplement: Supplementary file 2 [file 12951_2026_4663_MOESM1_ESM.docx]

**FIG7-A(HUVEC)**

**HUVEC-SIRT1-WB**

MOF818

MOF818@SEC

P-G^MOF818@SEC^

Model

Control

Sec

250kDa

37kDa

100kDa

150kDa

70kDa

50kDa

MOF818@SEC

P-G^MOF818@SEC^

**HUVEC-FOXO3a-WB**

MOF818

Control

Model

Sec

250kDa

100kDa

150kDa

37kDa

100kDa

70kDa

70kDa

50kDa

50kDa

MOF818@SEC

P-G^MOF818@SEC^

**HUVEC-SOD2-WB**

MOF818

Control

Model

Sec

40kDa

35kDa

25kDa

37kDa

25kDa

20kDa

20kDa

15kDa

15kDa

10kDa

**HUVEC-GAPDH-WB**

P-G^MOF818@SEC^

MOF818@SEC

MOF818

Model

Sec

Control

40kDa

37kDa

35kDa

25kDa

20kDa

10kDa

15kDa

**FIG7- B (L929)**

Control

Model

Sec

MOF818

MOF818@SEC

P-G^MOF818@SEC^

**L929-SIRT1-WB**

250kDa

37kDa

150kDa

100kDa

70kDa

50kDa

40kDa

35kDa

25kDa

20kDa

15kDa

**L929-FOXO3a-WB**

Control

Model

Sec

MOF818

MOF818@SEC

P-G^MOF818@SEC^

250kDa

150kDa

37kDa

100kDa

70kDa

50kDa

40kDa

35kDa

25kDa

20kDa

15kDa

**L929-SOD2-WB**

Control

Model

Sec

MOF818

MOF818@SEC

P-G^MOF818@SEC^

250kDa

100kDa

150kDa

70kDa

50kDa

40kDa

35kDa

37kDa

25kDa

20kDa

15kDa

**L929-GAPDH-WB**

Control

Model

Sec

MOF818

MOF818@SEC

P-G^MOF818@SEC^

250kDa

150kDa

100kDa

70kDa

50kDa

37kDa

40kDa

35kDa

25kDa

20kDa

15kDa

Control

Model

Sec

MOF818

MOF818@SEC

P-G^MOF818@SEC^

**FIG7- C (HaCAT)**

**HaCAT-SIRT1-WB**

250kDa

150kDa

100kDa

70kDa

50kDa

40kDa

35kDa

25kDa

20kDa

15kDa

37kDa

**HaCAT-FOXO3a-WB**

Control

Model

Sec

MOF818

MOF818@SEC

P-G^MOF818@SEC^

250kDa

150kDa

100kDa

70kDa

50kDa

40kDa

35kDa

25kDa

20kDa

15kDa

Control

Model

Sec

MOF818

MOF818@SEC

P-G^MOF818@SEC^

**HaCAT-SOD2-WB**

250kDa

150kDa

100kDa

70kDa

50kDa

40kDa

35kDa

25kDa

20kDa

15kDa

Control

Model

Sec

MOF818

MOF818@SEC

P-G^MOF818@SEC^

**HaCAT-GAPDH-WB**

250kDa

150kDa

100kDa

70kDa

50kDa

40kDa

35kDa

25kDa

20kDa

15kDa

**\**

**FIG7- G: Ubiquitination levels detected in HUVEC**

Control

Model

Sec

MOF818

MOF818@SEC

P-G^MOF818@SEC^

IP:SIRT1	UB

IGG

**Ubiquitin**

110kDa

IP:SIRT1	SIRT1

Control

Model

Sec

MOF818

MOF818@SEC

P-G^MOF818@SEC^

IGG

**SIRT1**

110kDa

**input**:SIRT1

Control

Model

Sec

MOF818

MOF818@SEC

P-G^MOF818@SEC^

**SIRT1**

110kDa

**input**:GAPDH

Control

Model

Sec

MOF818

MOF818@SEC

P-G^MOF818@SEC^

**GAPDH**

37kDa

**FIG7- H: Ubiquitination levels detected in L929**

Control

Model

Sec

MOF818

MOF818@SEC

P-G^MOF818@SEC^

IP:SIRT1	UB

IGG

**Ubiquitin**

110kDa

IP:SIRT1	SIRT1

Control

Model

Sec

MOF818

MOF818@SEC

P-G^MOF818@SEC^

IGG

**SIRT1**

110kDa

Control

Model

Sec

MOF818

MOF818@SEC

P-G^MOF818@SEC^

**input**:SIRT1

**SIRT1**

110kDa

**input**:GAPDH

Control

Model

Sec

MOF818

MOF818@SEC

P-G^MOF818@SEC^

**GAPDH**

37kDa

**FIG7- H: Ubiquitination levels detected in HaCAT**

Control

Model

Sec

MOF818

MOF818@SEC

P-G^MOF818@SEC^

IGG

IP:SIRT1	UB

**Ubiquitin**

110kDa

IP:SIRT1	SIRT1

Control

Model

Sec

MOF818

MOF818@SEC

P-G^MOF818@SEC^

**SIRT1**

110kDa

**input**:SIRT1

Control

Model

Sec

MOF818

MOF818@SEC

P-G^MOF818@SEC^

**SIRT1**

110kDa

Control

Model

Sec

MOF818

MOF818@SEC

P-G^MOF818@SEC^

**input**:GAPDH

**GAPDH**

37kDa

**FIG 9E sirt1**

**SIRT1**

100kDa

UB

**Ubiquitin**

100kDa

FOXO3a

**FOXO3a**

70kDa

SOD2

**SOD2**

25kDa

β-actin

**β-actin**

42kDa
